# Poly[[tetra­aqua-μ_4_-fumarato-di-μ_3_-fumarato-dineodymium(III)] trihydrate]

**DOI:** 10.1107/S1600536811046447

**Published:** 2011-11-09

**Authors:** Hong-ren Chen, Tian-sheng Tang, Jin Wang, Pei-lian Liu, Zeng Zhuo

**Affiliations:** a School of Chemistry and Environment, South China Normal University, Guangzhou 510006, People’s Republic of China; b Key Laboratory of Organofluorine Chemistry, Shanghai Institute of Organic Chemistry, Chinese Academy of Sciences, Shanghai 200032, People’s Republic of China

## Abstract

The title coordination polymer, {[Nd_2_(C_4_H_2_O_4_)_3_(H_2_O)_4_]·3H_2_O}, was synthesized by the reaction of neodymium(III) nitrate hexa­hydrate with fumaric acid in a water–methanol (7:3) solution. The asymmetric unit comprises two Nd^3+^ cations, three fumarate dianions (*L* ^2−^), four aqua ligands and three uncoordinated water mol­ecules. The carboxyl­ate groups of the fumarate dianions exhibit different coordination modes. In one fumarate dianion, two carboxyl­ate groups chelate two Nd^3+^ cations, while one of the O atoms is coordinated to another Nd^3+^ cation. Another fumarate dianion bridges three Nd^3+^ cations: one of the carboxyl­ate groups chelates one Nd^3+^ cation, while the other carboxyl­ate group bridges two Nd^3+^ cations in a monodentate mode. The third fumarate dianion bridges four Nd^3+^ cations, where one of the carboxyl­ate groups chelates one Nd^3+^ cation and coordinates in a monodentate mode to a second Nd^3+^, while the second carboxyl­ate groups bridges two Nd^3+^ cations in a monodentate mode and one O atom is coordinated to one Nd^3+^ cation. The Nd^3+^ cations are in a distorted tricapped–trigonal prismatic environment and coordinated by seven O atoms from the fumarate ligands and two O atoms from water mol­ecules. The Nd^3+^ cations are linked by two carboxyl­ate O atoms and two carboxyl­ate groups, generating infinite Nd–O chains to form a three-dimensional framework. There are O—H⋯O and C—H⋯O hydrogen-bonding interactions between the coordin­ated and uncoordinated water mol­ecules and carboxyl­ate O atoms.

## Related literature

For applications of metal complexes with carboxylato ligands, see: Eliseeva *et al.* (2010 ▶); Kim *et al.* (2001 ▶); Seki & Mori (2002 ▶). 
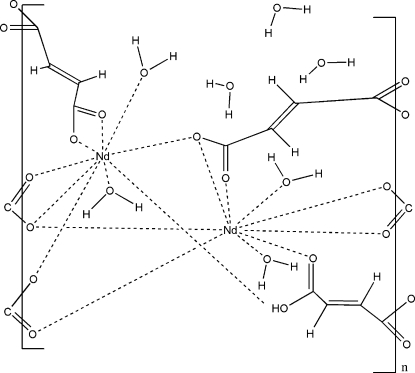


## Experimental

### 

#### Crystal data

[Nd_2_(C_4_H_2_O_4_)_3_(H_2_O)_4_]·3H_2_O
*M* *_r_* = 756.76
Monoclinic, 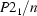

*a* = 9.5810 (9) Å
*b* = 14.8675 (15) Å
*c* = 14.9056 (14) Å
β = 91.538 (5)°
*V* = 2122.5 (4) Å^3^
*Z* = 4
Mo *K*α radiation
μ = 4.93 mm^−1^
*T* = 298 K
0.16 × 0.15 × 0.14 mm

#### Data collection

Bruker APEXII CCD diffractometer
Absorption correction: multi-scan (*SADABS*; Sheldrick, 1996 ▶) *T*_min_ = 0.459, *T*_max_ = 0.501
24284 measured reflections
5150 independent reflections
4060 reflections with *I* > 2σ(*I*)
*R*_int_ = 0.052

#### Refinement

*R*[*F* ^2^ > 2σ(*F* ^2^)] = 0.030
*wR*(*F* ^2^) = 0.075
*S* = 1.05
5150 reflections
306 parameters
H atoms treated by a mixture of independent and constrained refinement
Δρ_max_ = 1.36 e Å^−3^
Δρ_min_ = −0.89 e Å^−3^

### 

Data collection: *APEX2* (Bruker, 2008 ▶); cell refinement: *SAINT* (Bruker, 2008 ▶); data reduction: *SAINT*; program(s) used to solve structure: *SHELXS97* (Sheldrick, 2008 ▶); program(s) used to refine structure: *SHELXL97* (Sheldrick, 2008 ▶); molecular graphics: *SHELXTL* (Sheldrick, 2008 ▶); software used to prepare material for publication: *SHELXTL*.

## Supplementary Material

Crystal structure: contains datablock(s) global, I. DOI: 10.1107/S1600536811046447/ez2264sup1.cif

Structure factors: contains datablock(s) I. DOI: 10.1107/S1600536811046447/ez2264Isup2.hkl

Additional supplementary materials:  crystallographic information; 3D view; checkCIF report

## Figures and Tables

**Table 1:** Hydrogen-bond geometry (Å, °)

*D*—H⋯*A*	*D*—H	H⋯*A*	*D*⋯*A*	*D*—H⋯*A*
O2*W*—H2*WA*⋯O1	0.85	2.57	3.103 (13)	122
O2*W*—H2*WA*⋯O3	0.85	2.52	3.319 (13)	158
O2*W*—H2*WB*⋯O1*W*^i^	0.85	2.53	2.98 (2)	114
O3*W*—H3*WD*⋯O24^i^	0.85	2.07	2.896 (6)	165
O3*W*—H3*WC*⋯O1*W*	0.85	2.12	2.60 (2)	115
O3*W*—H3*WC*⋯O2*W*	0.85	2.08	2.911 (13)	165
O1*W*—H1*WD*⋯O2*W*	0.85	2.06	2.634 (19)	124
O1*W*—H1*WC*⋯O6^ii^	0.85	2.11	2.959 (17)	178
O8—H8*C*⋯O3*W*^iii^	0.85	2.05	2.829 (5)	152
O8—H8*B*⋯O1^iv^	0.85	1.91	2.745 (5)	169
O13—H13*A*⋯O3*W*^iii^	0.85	2.14	2.938 (6)	157
O13—H13*B*⋯O25^v^	0.82	2.02	2.787 (5)	157
O14—H14*A*⋯O12^vi^	0.86 (6)	1.88 (6)	2.740 (5)	172 (6)
O14—H14*B*⋯O4^iii^	0.75 (5)	2.04 (6)	2.776 (5)	166 (6)
O16—H16*A*⋯O27^vii^	0.72	2.02	2.714 (5)	160
O16—H16*C*⋯O2^viii^	0.85	2.07	2.915 (5)	171
C3—H3⋯O24^v^	0.93	2.53	3.345 (6)	147
C8—H8⋯O12^iv^	0.93	2.58	3.417 (6)	150

Symmetry codes: (i) 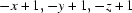; (ii) 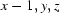; (iii) 
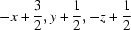
; (iv) 
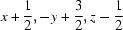
; (v) 
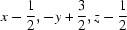
; (vi) 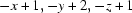; (vii) 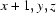; (viii) 
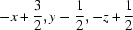
.

**Fig. 1.**
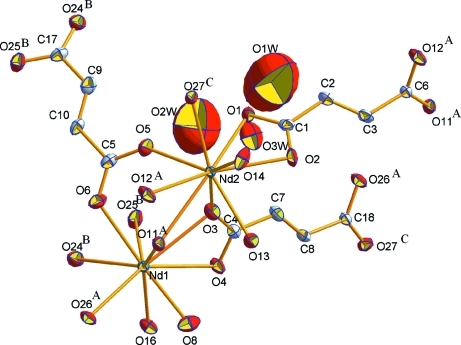
View of the local coordination sphere around the neodymium(III) centers with the atom-numbering scheme. Displacement ellipsoids are drawn at the 50% probability level. [Symmetry codes: (A)1 + x,y,z; (B)2 - x,1 - y,1 - z; (C)1/2 + x,3/2 - y,1/2 + z.]

**Fig. 2.**
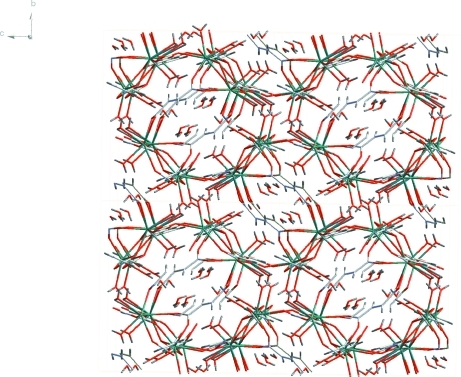
Perspective view of the crystal packing.

## supplementary crystallographic information

### Comment

Recently, many metal complexes of carboxylates and lanthanide complexes which display interesting properties have been reported: Mn dicarboxylate compounds present antiferromagnetic interactions (Kim *et al.*, 2001), while Cu dicarboxylates have uniform micropores, high porosities and gas adsorption capacities (Seki *et al.*, 2002). In addition, lanthanide complexes can be used as active materials in luminescent devices (Eliseeva *et al.*, 2010). In this paper, we report the title complex, obtained by the reaction of neodymium(III) nitrate hexahydrate with fumaric acid in a water-methanol (7:3) solution.

The structure of the asymmetric unit of the title complex is shown in Fig. 1. It comprises two Nd^3+^ cations, three fumarate dianions (*L*^2-^), four aqua ligands and three uncoordinated water molecules. The carboxylate groups of the fumarate dianion exhibit different coordination modes. In one fumarate dianion two carboxylate groups chelate with two Nd^3+^ cations, while one of the O atoms (O11) is coordinated with another Nd^3+^ cation. The second fumarate dianion bridges three Nd^3+^ cations, one of carboxylate groups chelating with one Nd^3+^ cation and the other carboxylate groups bridging two Nd^3+^ cations in monodentate mode. The third fumarate ligand bridges four Nd^3+^ cations, one of carboxylate groups chelating with one Nd^3+^ cation and one of carboxylate groups bridging two Nd^3+^ cations in monodentate mode, while one O atom (O3) is coordinated with a third Nd^3+^ cation. The Nd^3+^ cations are situated within a distorted tricapped trigonal prism and coordinated by seven O atoms from the fumarate dianion ligands and two O atom from water molecules. The Nd—O bond distances range from 2.387 (3) to 2.655 (3) Å. The O—Nd—O bond angles range from 73.4 (1) to 155.0 (1)°. The Nd^3+^ cations are linked by two carboxylate O atoms (O3 and O11) and two carboxylate groups (O5—C5—O6 and O26—C18—O27) to generate infinite neodymium-oxygen chains (Fig. 2). The chains are further connected by the ligands to form a three-dimensional framework. The crystal is stabilized by hydrogen bond interactions between the coordinated and uncoordinated water molecules and the carboxylate O atoms (Table 1).

### Experimental

Fumaric acid (0.3 mmol, 0.035 g) and neodymium(III) nitrate hexahydrate(0.5 mmol, 0.22 g) were dissolved in a water-methanol(7:3) solution (10 ml). The mixture was transferred to a 20 ml Teflon-lined stainless steel autoclave, which was heated at 443 K for 96 h. The reactor was cooled to room temperature over a period of 24 h. Green crystals were obtained after filtration, washing with water and vacum drying.

### Refinement

Carbon-bound H atoms were included in the riding-model approximation, with C—H =0.93Å and with *U*_iso_(H) = 1.2*U*_eq_(C). The H atoms of the water molecules were located in Fourier difference maps and allowed to ride on their parent atoms with *U*_iso_(H) = 1.2*U*_eq_(O).

### Crystal data

**Table:**

[Nd_2_(C_4_H_2_O_4_)_3_(H_2_O)_4_]·3H_2_O	*F*(000) = 1456.0
*M**_r_* = 756.76	*D*_x_ = 2.368 Mg m^−^^3^
Monoclinic, *P*2_1_/*n*	Mo *K*α radiation, λ = 0.71073 Å
Hall symbol: -P 2yn	Cell parameters from 6284 reflections
*a* = 9.5810 (9) Å	θ = 2.5–28.0°
*b* = 14.8675 (15) Å	µ = 4.93 mm^−^^1^
*c* = 14.9056 (14) Å	*T* = 298 K
β = 91.538 (5)°	Block, green
*V* = 2122.5 (4) Å^3^	0.16 × 0.15 × 0.14 mm
*Z* = 4	

### Data collection

**Table:**

Bruker APEXII CCD diffractometer	5150 independent reflections
Radiation source: fine-focus sealed tube	4060 reflections with *I* > 2σ(*I*)
graphite	*R*_int_ = 0.052
phi and ω scans	θ_max_ = 28.1°, θ_min_ = 1.9°
Absorption correction: multi-scan (*SADABS*; Sheldrick, 1996)	*h* = −12→12
*T*_min_ = 0.459, *T*_max_ = 0.501	*k* = −19→19
24284 measured reflections	*l* = −15→19

### Refinement

**Table:**

Refinement on *F*^2^	Primary atom site location: structure-invariant direct methods
Least-squares matrix: full	Secondary atom site location: difference Fourier map
*R*[*F*^2^ > 2σ(*F*^2^)] = 0.030	Hydrogen site location: inferred from neighbouring sites
*wR*(*F*^2^) = 0.075	H atoms treated by a mixture of independent and constrained refinement
*S* = 1.05	*w* = 1/[σ^2^(*F*_o_^2^) + (0.0302*P*)^2^ + 2.6259*P*] where *P* = (*F*_o_^2^ + 2*F*_c_^2^)/3
5150 reflections	(Δ/σ)_max_ = 0.002
306 parameters	Δρ_max_ = 1.36 e Å^−^^3^
0 restraints	Δρ_min_ = −0.89 e Å^−^^3^

### Special details

**Table:**

Geometry. All e.s.d.'s (except the e.s.d. in the dihedral angle between two l.s. planes) are estimated using the full covariance matrix. The cell e.s.d.'s are taken into account individually in the estimation of e.s.d.'s in distances, angles and torsion angles; correlations between e.s.d.'s in cell parameters are only used when they are defined by crystal symmetry. An approximate (isotropic) treatment of cell e.s.d.'s is used for estimating e.s.d.'s involving l.s. planes.
Refinement. Refinement of *F*^2^ against ALL reflections. The weighted *R*-factor *wR* and goodness of fit *S* are based on *F*^2^, conventional *R*-factors *R* are based on *F*, with *F* set to zero for negative *F*^2^. The threshold expression of *F*^2^ > σ(*F*^2^) is used only for calculating *R*-factors(gt) *etc*. and is not relevant to the choice of reflections for refinement. *R*-factors based on *F*^2^ are statistically about twice as large as those based on *F*, and *R*- factors based on ALL data will be even larger.

### Fractional atomic coordinates and isotropic or equivalent isotropic displacement parameters (Å^2^)

**Table:**

	*x*	*y*	*z*	*U*_iso_*/*U*_eq_	
Nd1	1.02953 (3)	0.663992 (15)	0.246217 (16)	0.01439 (7)	
Nd2	0.80991 (2)	0.846421 (15)	0.405801 (16)	0.01358 (7)	
O1	0.5842 (3)	0.7709 (2)	0.4569 (2)	0.0234 (8)	
C3	0.2687 (5)	0.8568 (3)	0.3655 (3)	0.0227 (11)	
H3	0.3058	0.8783	0.3127	0.027*	
O2	0.5619 (3)	0.8871 (2)	0.3665 (2)	0.0264 (8)	
C1	0.5090 (5)	0.8282 (3)	0.4166 (3)	0.0189 (10)	
C2	0.3555 (5)	0.8281 (4)	0.4280 (4)	0.0291 (12)	
H2	0.3202	0.8068	0.4815	0.035*	
O3	0.7836 (3)	0.7226 (2)	0.2985 (2)	0.0248 (8)	
O4	0.7899 (3)	0.6470 (2)	0.1732 (2)	0.0241 (8)	
C4	0.7199 (5)	0.6861 (3)	0.2319 (3)	0.0186 (10)	
O6	1.0906 (4)	0.6561 (2)	0.4082 (2)	0.0288 (8)	
C5	1.0122 (6)	0.6644 (3)	0.4739 (3)	0.0215 (11)	
O5	0.9116 (4)	0.7183 (2)	0.4785 (2)	0.0312 (9)	
O8	1.0189 (4)	0.7802 (2)	0.1277 (2)	0.0342 (9)	
H8B	1.0470	0.7594	0.0782	0.041*	
H8C	1.0621	0.8282	0.1422	0.041*	
C8	0.4972 (5)	0.6643 (3)	0.1528 (3)	0.0229 (11)	
H8	0.5490	0.6512	0.1025	0.028*	
C6	0.1155 (5)	0.8573 (3)	0.3732 (3)	0.0167 (10)	
C7	0.5665 (5)	0.6870 (3)	0.2248 (3)	0.0249 (11)	
H7	0.5169	0.7049	0.2745	0.030*	
C10	1.0375 (5)	0.6029 (3)	0.5517 (3)	0.0238 (11)	
H10	1.1223	0.5725	0.5560	0.029*	
C9	0.9469 (6)	0.5895 (3)	0.6139 (3)	0.0284 (12)	
H9	0.8705	0.6280	0.6170	0.034*	
O11	0.0390 (3)	0.8180 (2)	0.3136 (2)	0.0191 (7)	
O12	0.0605 (3)	0.8974 (2)	0.4368 (2)	0.0283 (8)	
O14	0.7818 (4)	1.0060 (2)	0.4411 (3)	0.0310 (9)	
O13	0.8092 (4)	0.9288 (3)	0.2613 (2)	0.0359 (9)	
H13A	0.8857	0.9226	0.2344	0.043*	
H13B	0.7472	0.9489	0.2284	0.043*	
O16	1.0338 (3)	0.5710 (2)	0.1065 (2)	0.0244 (8)	
H16C	1.0155	0.5158	0.1155	0.029*	
H16A	1.1004	0.5787	0.0861	0.029*	
C17	0.9609 (6)	0.5154 (3)	0.6799 (3)	0.0229 (11)	
O25	1.0774 (4)	0.4770 (2)	0.6945 (2)	0.0268 (8)	
O24	0.8508 (4)	0.4865 (2)	0.7148 (2)	0.0280 (8)	
C18	0.3435 (5)	0.6570 (3)	0.1423 (3)	0.0172 (10)	
O26	0.2692 (3)	0.6793 (2)	0.2068 (2)	0.0264 (8)	
O27	0.2955 (3)	0.6265 (2)	0.0686 (2)	0.0234 (8)	
O2W	0.6477 (13)	0.5680 (8)	0.4310 (8)	0.236 (5)	
H2WA	0.6803	0.6169	0.4106	0.354*	
H2WB	0.7017	0.5507	0.4737	0.354*	
O3W	0.4332 (5)	0.4644 (3)	0.3352 (3)	0.0568 (13)	
H3WC	0.4845	0.5021	0.3634	0.085*	
H3WD	0.3534	0.4878	0.3252	0.085*	
H14A	0.833 (6)	1.032 (4)	0.482 (4)	0.043 (19)*	
H14B	0.776 (6)	1.044 (4)	0.408 (4)	0.023 (16)*	
O1W	0.3772 (16)	0.5909 (13)	0.4489 (10)	0.342 (10)	
H1WD	0.4418	0.6067	0.4142	0.514*	
H1WC	0.2950	0.6105	0.4385	0.514*	

### Atomic displacement parameters (Å^2^)

**Table:**

	*U*^11^	*U*^22^	*U*^33^	*U*^12^	*U*^13^	*U*^23^
Nd1	0.01165 (14)	0.01670 (12)	0.01485 (13)	−0.00010 (9)	0.00092 (10)	−0.00118 (9)
Nd2	0.00971 (14)	0.01650 (12)	0.01448 (13)	0.00087 (9)	−0.00055 (9)	−0.00120 (9)
O1	0.0173 (19)	0.0271 (18)	0.0256 (19)	0.0033 (15)	−0.0008 (14)	0.0066 (15)
C3	0.016 (3)	0.030 (3)	0.022 (3)	−0.005 (2)	0.006 (2)	0.002 (2)
O2	0.0102 (18)	0.0298 (19)	0.039 (2)	0.0005 (14)	−0.0017 (15)	0.0124 (16)
C1	0.019 (3)	0.025 (2)	0.013 (2)	−0.003 (2)	0.0025 (19)	−0.0046 (18)
C2	0.016 (3)	0.045 (3)	0.026 (3)	0.001 (2)	0.006 (2)	0.009 (2)
O3	0.0194 (19)	0.0286 (18)	0.0262 (19)	−0.0038 (15)	−0.0014 (15)	−0.0119 (15)
O4	0.0141 (18)	0.0347 (19)	0.0235 (19)	−0.0003 (15)	−0.0001 (14)	−0.0118 (15)
C4	0.016 (3)	0.017 (2)	0.022 (3)	0.0004 (19)	0.002 (2)	0.0004 (19)
O6	0.040 (2)	0.0298 (19)	0.0168 (18)	0.0040 (16)	0.0007 (16)	0.0004 (14)
C5	0.031 (3)	0.017 (2)	0.016 (2)	0.000 (2)	−0.002 (2)	0.0009 (18)
O5	0.034 (2)	0.0296 (19)	0.030 (2)	0.0101 (17)	0.0015 (17)	0.0109 (16)
O8	0.052 (3)	0.0264 (19)	0.024 (2)	−0.0025 (18)	0.0027 (18)	−0.0020 (16)
C8	0.016 (3)	0.034 (3)	0.019 (3)	0.004 (2)	0.005 (2)	−0.002 (2)
C6	0.013 (2)	0.020 (2)	0.017 (2)	0.0006 (19)	0.0011 (19)	0.0031 (18)
C7	0.015 (3)	0.034 (3)	0.025 (3)	0.001 (2)	0.002 (2)	−0.011 (2)
C10	0.029 (3)	0.021 (2)	0.021 (3)	0.005 (2)	−0.005 (2)	0.004 (2)
C9	0.035 (3)	0.021 (2)	0.030 (3)	0.009 (2)	0.001 (2)	0.006 (2)
O11	0.0153 (18)	0.0236 (16)	0.0183 (17)	−0.0007 (14)	−0.0018 (14)	−0.0039 (13)
O12	0.0138 (18)	0.041 (2)	0.031 (2)	−0.0051 (16)	0.0035 (15)	−0.0138 (17)
O14	0.043 (3)	0.0159 (18)	0.033 (2)	0.0024 (17)	−0.0169 (19)	−0.0004 (17)
O13	0.029 (2)	0.049 (2)	0.030 (2)	0.0144 (19)	0.0089 (17)	0.0166 (18)
O16	0.0199 (19)	0.0240 (17)	0.030 (2)	−0.0037 (15)	0.0067 (15)	−0.0051 (15)
C17	0.033 (3)	0.018 (2)	0.018 (2)	0.000 (2)	0.000 (2)	0.0011 (19)
O25	0.025 (2)	0.0219 (17)	0.033 (2)	−0.0031 (15)	−0.0052 (16)	0.0079 (15)
O24	0.030 (2)	0.0263 (18)	0.028 (2)	0.0038 (16)	0.0063 (16)	0.0026 (15)
C18	0.011 (2)	0.020 (2)	0.020 (2)	0.0007 (18)	0.0020 (19)	0.0015 (18)
O26	0.0138 (19)	0.042 (2)	0.0239 (19)	−0.0025 (15)	0.0050 (15)	−0.0083 (16)
O27	0.0188 (19)	0.0361 (19)	0.0151 (17)	−0.0072 (15)	−0.0027 (14)	0.0018 (14)
O2W	0.251 (14)	0.170 (10)	0.285 (15)	−0.001 (10)	−0.021 (11)	0.011 (10)
O3W	0.054 (3)	0.040 (3)	0.076 (4)	0.005 (2)	−0.001 (3)	−0.011 (2)
O1W	0.264 (16)	0.44 (3)	0.32 (2)	−0.100 (17)	−0.051 (14)	0.098 (19)

### Geometric parameters (Å, °)

**Table:**

Nd1—O26^i^	2.397 (3)	O8—H8C	0.8501
Nd1—O8	2.471 (3)	C8—C7	1.291 (7)
Nd1—O6	2.473 (3)	C8—C18	1.480 (7)
Nd1—O11^i^	2.501 (3)	C8—H8	0.9300
Nd1—O16	2.502 (3)	C6—O12	1.249 (5)
Nd1—O25^ii^	2.504 (3)	C6—O11	1.276 (5)
Nd1—O4	2.527 (3)	C6—Nd2^iv^	2.985 (5)
Nd1—O4	2.527 (3)	C7—H7	0.9300
Nd1—O24^ii^	2.573 (3)	C10—C9	1.302 (7)
Nd1—O3	2.649 (3)	C10—H10	0.9300
Nd1—O3	2.649 (3)	C9—C17	1.482 (6)
Nd1—C17^ii^	2.886 (5)	C9—H9	0.9300
Nd2—O5	2.387 (3)	O11—Nd1^iv^	2.501 (3)
Nd2—O14	2.446 (4)	O11—Nd2^iv^	2.655 (3)
Nd2—O3	2.447 (3)	O12—Nd2^iv^	2.548 (3)
Nd2—O3	2.447 (3)	O14—H14A	0.86 (6)
Nd2—O27^iii^	2.467 (3)	O14—H14B	0.75 (5)
Nd2—O13	2.477 (3)	O13—H13A	0.8499
Nd2—O2	2.507 (3)	O13—H13B	0.8175
Nd2—O12^i^	2.548 (3)	O16—H16C	0.8499
Nd2—O1	2.570 (3)	O16—H16A	0.7231
Nd2—O11^i^	2.655 (3)	C17—O24	1.265 (6)
Nd2—C6^i^	2.985 (5)	C17—O25	1.267 (6)
O1—C1	1.258 (5)	C17—Nd1^ii^	2.886 (5)
C3—C2	1.304 (7)	O25—Nd1^ii^	2.504 (3)
C3—C6	1.475 (7)	O24—Nd1^ii^	2.573 (3)
C3—H3	0.9300	C18—O26	1.257 (5)
O2—C1	1.266 (5)	C18—O27	1.264 (6)
C1—C2	1.484 (7)	O26—Nd1^iv^	2.397 (3)
C2—H2	0.9300	O27—Nd2^v^	2.467 (3)
O3—O3	0.000 (7)	O2W—O2W	0.00 (2)
O3—C4	1.274 (5)	O2W—O2W	0.00 (2)
O4—O4	0.000 (7)	O2W—H2WA	0.8500
O4—C4	1.259 (5)	O2W—H2WB	0.8499
C4—O4	1.259 (5)	O3W—O3W	0.000 (16)
C4—O3	1.274 (5)	O3W—H3WC	0.8500
C4—C7	1.471 (7)	O3W—H3WD	0.8500
O6—C5	1.256 (6)	O1W—O1W	0.00 (4)
C5—O5	1.256 (6)	O1W—H1WD	0.8500
C5—C10	1.492 (6)	O1W—H1WC	0.8502
O8—H8B	0.8500		
			
O26^i^—Nd1—O8	77.26 (12)	O2—Nd2—O11^i^	135.19 (11)
O26^i^—Nd1—O6	92.32 (12)	O12^i^—Nd2—O11^i^	49.67 (10)
O8—Nd1—O6	137.38 (11)	O1—Nd2—O11^i^	142.94 (10)
O26^i^—Nd1—O11^i^	89.22 (11)	O5—Nd2—C6^i^	74.15 (12)
O8—Nd1—O11^i^	69.37 (11)	O14—Nd2—C6^i^	95.55 (13)
O6—Nd1—O11^i^	69.27 (11)	O3—Nd2—C6^i^	91.06 (12)
O26^i^—Nd1—O16	79.13 (11)	O3—Nd2—C6^i^	91.06 (12)
O8—Nd1—O16	78.01 (11)	O27^iii^—Nd2—C6^i^	103.44 (12)
O6—Nd1—O16	141.11 (11)	O13—Nd2—C6^i^	79.12 (12)
O11^i^—Nd1—O16	147.08 (11)	O2—Nd2—C6^i^	151.25 (12)
O26^i^—Nd1—O25^ii^	124.75 (12)	O12^i^—Nd2—C6^i^	24.49 (11)
O8—Nd1—O25^ii^	145.90 (12)	O1—Nd2—C6^i^	155.32 (11)
O6—Nd1—O25^ii^	72.77 (11)	O11^i^—Nd2—C6^i^	25.30 (11)
O11^i^—Nd1—O25^ii^	129.43 (11)	C1—O1—Nd2	92.2 (3)
O16—Nd1—O25^ii^	81.22 (11)	C2—C3—C6	124.3 (5)
O26^i^—Nd1—O4	140.31 (11)	C2—C3—H3	117.8
O8—Nd1—O4	75.09 (12)	C6—C3—H3	117.8
O6—Nd1—O4	127.20 (12)	C1—O2—Nd2	95.0 (3)
O11^i^—Nd1—O4	106.71 (10)	O1—C1—O2	121.1 (4)
O16—Nd1—O4	67.72 (10)	O1—C1—C2	120.1 (4)
O25^ii^—Nd1—O4	72.11 (11)	O2—C1—C2	118.8 (4)
O26^i^—Nd1—O4	140.31 (11)	C3—C2—C1	122.2 (5)
O8—Nd1—O4	75.09 (12)	C3—C2—H2	118.9
O6—Nd1—O4	127.20 (12)	C1—C2—H2	118.9
O11^i^—Nd1—O4	106.71 (10)	O3—O3—C4	0(10)
O16—Nd1—O4	67.72 (10)	O3—O3—Nd2	0(10)
O25^ii^—Nd1—O4	72.11 (11)	C4—O3—Nd2	150.4 (3)
O4—Nd1—O4	0.00 (16)	O3—O3—Nd1	0(6)
O26^i^—Nd1—O24^ii^	73.38 (12)	C4—O3—Nd1	92.4 (3)
O8—Nd1—O24^ii^	141.13 (11)	Nd2—O3—Nd1	111.28 (12)
O6—Nd1—O24^ii^	69.25 (11)	O4—O4—C4	0(10)
O11^i^—Nd1—O24^ii^	133.95 (11)	O4—O4—Nd1	0(3)
O16—Nd1—O24^ii^	71.95 (11)	C4—O4—Nd1	98.6 (3)
O25^ii^—Nd1—O24^ii^	51.45 (11)	O4—C4—O4	0.0 (3)
O4—Nd1—O24^ii^	113.85 (11)	O4—C4—O3	119.0 (4)
O4—Nd1—O24^ii^	113.85 (11)	O4—C4—O3	119.0 (4)
O26^i^—Nd1—O3	155.04 (11)	O4—C4—O3	119.0 (4)
O8—Nd1—O3	87.75 (11)	O4—C4—O3	119.0 (4)
O6—Nd1—O3	85.27 (11)	O3—C4—O3	0.0 (3)
O11^i^—Nd1—O3	66.66 (10)	O4—C4—C7	120.3 (4)
O16—Nd1—O3	117.47 (10)	O4—C4—C7	120.3 (4)
O25^ii^—Nd1—O3	78.22 (11)	O3—C4—C7	120.6 (4)
O4—Nd1—O3	49.80 (10)	O3—C4—C7	120.6 (4)
O4—Nd1—O3	49.80 (10)	C5—O6—Nd1	128.7 (3)
O24^ii^—Nd1—O3	127.89 (11)	O6—C5—O5	125.6 (4)
O26^i^—Nd1—O3	155.04 (11)	O6—C5—C10	117.2 (4)
O8—Nd1—O3	87.75 (11)	O5—C5—C10	117.2 (4)
O6—Nd1—O3	85.27 (11)	C5—O5—Nd2	142.0 (3)
O11^i^—Nd1—O3	66.66 (10)	Nd1—O8—H8B	111.0
O16—Nd1—O3	117.47 (10)	Nd1—O8—H8C	113.2
O25^ii^—Nd1—O3	78.22 (11)	H8B—O8—H8C	111.4
O4—Nd1—O3	49.80 (10)	C7—C8—C18	126.5 (5)
O4—Nd1—O3	49.80 (10)	C7—C8—H8	116.8
O24^ii^—Nd1—O3	127.89 (11)	C18—C8—H8	116.8
O3—Nd1—O3	0.00 (6)	O12—C6—O11	120.1 (4)
O26^i^—Nd1—C17^ii^	99.25 (14)	O12—C6—C3	120.1 (4)
O8—Nd1—C17^ii^	156.81 (12)	O11—C6—C3	119.8 (4)
O6—Nd1—C17^ii^	65.09 (12)	O12—C6—Nd2^iv^	57.8 (2)
O11^i^—Nd1—C17^ii^	133.80 (12)	O11—C6—Nd2^iv^	62.7 (2)
O16—Nd1—C17^ii^	78.82 (12)	C3—C6—Nd2^iv^	174.0 (3)
O25^ii^—Nd1—C17^ii^	25.97 (12)	C8—C7—C4	123.2 (5)
O4—Nd1—C17^ii^	95.22 (13)	C8—C7—H7	118.4
O4—Nd1—C17^ii^	95.22 (13)	C4—C7—H7	118.4
O24^ii^—Nd1—C17^ii^	25.99 (12)	C9—C10—C5	123.3 (5)
O3—Nd1—C17^ii^	102.18 (13)	C9—C10—H10	118.3
O3—Nd1—C17^ii^	102.18 (13)	C5—C10—H10	118.3
O5—Nd2—O14	136.23 (13)	C10—C9—C17	122.4 (5)
O5—Nd2—O3	74.35 (12)	C10—C9—H9	118.8
O14—Nd2—O3	149.32 (13)	C17—C9—H9	118.8
O5—Nd2—O3	74.35 (12)	C6—O11—Nd1^iv^	135.4 (3)
O14—Nd2—O3	149.32 (13)	C6—O11—Nd2^iv^	92.0 (3)
O3—Nd2—O3	0.00 (10)	Nd1^iv^—O11—Nd2^iv^	109.38 (11)
O5—Nd2—O27^iii^	73.51 (12)	C6—O12—Nd2^iv^	97.7 (3)
O14—Nd2—O27^iii^	67.73 (13)	Nd2—O14—H14A	121 (4)
O3—Nd2—O27^iii^	139.28 (12)	Nd2—O14—H14B	127 (4)
O3—Nd2—O27^iii^	139.28 (12)	H14A—O14—H14B	100 (5)
O5—Nd2—O13	141.36 (12)	Nd2—O13—H13A	112.0
O14—Nd2—O13	73.12 (14)	Nd2—O13—H13B	133.5
O3—Nd2—O13	78.82 (12)	H13A—O13—H13B	112.2
O3—Nd2—O13	78.82 (12)	Nd1—O16—H16C	113.1
O27^iii^—Nd2—O13	140.84 (12)	Nd1—O16—H16A	107.2
O5—Nd2—O2	132.21 (12)	H16C—O16—H16A	114.1
O14—Nd2—O2	72.98 (13)	O24—C17—O25	121.1 (4)
O3—Nd2—O2	87.02 (12)	O24—C17—C9	117.7 (5)
O3—Nd2—O2	87.02 (12)	O25—C17—C9	120.9 (5)
O27^iii^—Nd2—O2	96.50 (11)	O24—C17—Nd1^ii^	63.1 (2)
O13—Nd2—O2	72.36 (11)	O25—C17—Nd1^ii^	60.0 (2)
O5—Nd2—O12^i^	77.49 (12)	C9—C17—Nd1^ii^	160.5 (3)
O14—Nd2—O12^i^	77.37 (13)	C17—O25—Nd1^ii^	94.1 (3)
O3—Nd2—O12^i^	114.97 (11)	C17—O24—Nd1^ii^	91.0 (3)
O3—Nd2—O12^i^	114.97 (11)	O26—C18—O27	124.1 (4)
O27^iii^—Nd2—O12^i^	81.39 (11)	O26—C18—C8	118.8 (4)
O13—Nd2—O12^i^	89.50 (12)	O27—C18—C8	117.1 (4)
O2—Nd2—O12^i^	148.62 (12)	C18—O26—Nd1^iv^	136.8 (3)
O5—Nd2—O1	81.57 (11)	C18—O27—Nd2^v^	140.2 (3)
O14—Nd2—O1	105.16 (13)	O2W—O2W—O2W	0(10)
O3—Nd2—O1	78.02 (11)	O2W—O2W—H2WA	0.0
O3—Nd2—O1	78.02 (11)	O2W—O2W—H2WA	0.0
O27^iii^—Nd2—O1	73.15 (11)	O2W—O2W—H2WB	0.0
O13—Nd2—O1	119.40 (12)	O2W—O2W—H2WB	0.0
O2—Nd2—O1	51.29 (10)	H2WA—O2W—H2WB	107.7
O12^i^—Nd2—O1	150.73 (11)	O3W—O3W—H3WC	0.0
O5—Nd2—O11^i^	76.98 (11)	O3W—O3W—H3WD	0.0
O14—Nd2—O11^i^	111.28 (12)	H3WC—O3W—H3WD	108.7
O3—Nd2—O11^i^	67.31 (10)	O1W—O1W—H1WD	0.0
O3—Nd2—O11^i^	67.31 (10)	O1W—O1W—H1WC	0.0
O27^iii^—Nd2—O11^i^	127.13 (10)	H1WD—O1W—H1WC	118.8
O13—Nd2—O11^i^	67.16 (11)		

Symmetry codes: (i) *x*+1, *y*, *z*; (ii) −*x*+2, −*y*+1, −*z*+1;  (iii) *x*+1/2, −*y*+3/2, *z*+1/2; (iv) *x*−1, *y*, *z*; (v) *x*−1/2, −*y*+3/2, *z*−1/2.

### Hydrogen-bond geometry (Å, °)

**Table:**

*D*—H···*A*	*D*—H	H···*A*	*D*···*A*	*D*—H···*A*
O2W—H2WA···O1	0.85	2.57	3.103 (13)	122.
O2W—H2WA···O3	0.85	2.52	3.319 (13)	158.
O2W—H2WB···O1W^vi^	0.85	2.53	2.98 (2)	114.
O3W—H3WD···O24^vi^	0.85	2.07	2.896 (6)	165.
O3W—H3WC···O1W	0.85	2.12	2.60 (2)	115.
O3W—H3WC···O2W	0.85	2.08	2.911 (13)	165.
O1W—H1WD···O2W	0.85	2.06	2.634 (19)	124.
O1W—H1WC···O6^iv^	0.85	2.11	2.959 (17)	178.
O8—H8C···O3W^vii^	0.85	2.05	2.829 (5)	152.
O8—H8B···O1^viii^	0.85	1.91	2.745 (5)	169.
O13—H13A···O3W^vii^	0.85	2.14	2.938 (6)	157.
O13—H13B···O25^v^	0.82	2.02	2.787 (5)	157.
O14—H14A···O12^ix^	0.86 (6)	1.88 (6)	2.740 (5)	172 (6)
O14—H14B···O4^vii^	0.75 (5)	2.04 (6)	2.776 (5)	166 (6)
O16—H16A···O27^i^	0.72	2.02	2.714 (5)	160.
O16—H16C···O2^x^	0.85	2.07	2.915 (5)	171.
C3—H3···O24^v^	0.93	2.53	3.345 (6)	147.
C8—H8···O12^viii^	0.93	2.58	3.417 (6)	150.

Symmetry codes:  (vi) −*x*+1, −*y*+1, −*z*+1; (iv) *x*−1, *y*, *z*; (vii) −*x*+3/2, *y*+1/2, −*z*+1/2; (viii) *x*+1/2, −*y*+3/2, *z*−1/2; (v) *x*−1/2, −*y*+3/2, *z*−1/2; (ix) −*x*+1, −*y*+2, −*z*+1; (i) *x*+1, *y*, *z*; (x) −*x*+3/2, *y*−1/2, −*z*+1/2.
